# Synthesis, crystal structure, Hirshfeld surface analysis, DFT and NBO study of ethyl 1-(4-fluoro­phen­yl)-4-[(4-fluoro­phen­yl)amino]-2,6-diphenyl-1,2,5,6-tetra­hydro­pyridine-3-carboxyl­ate

**DOI:** 10.1107/S205698902300748X

**Published:** 2023-09-08

**Authors:** Ravi Bansal, Ray J. Butcher, Sushil K. Gupta

**Affiliations:** a School of Studies in Chemistry, Jiwaji University, Gwalior 474011, India; b Department of Chemistry, Howard University, 525 College Street NW, Washington, DC 20059, USA; University of Aberdeen, United Kingdom

**Keywords:** Functionalized tetra­hydro­pyridine, crystal structure, Hirshfeld surface analysis, two-dimensional fingerprint plot, DFT, NBO

## Abstract

The title com­pound, C_32_H_28_F_2_N_2_O_2_, a highly functionalized tetra­hydro­pyridine, was synthesized by a one-pot multi-com­ponent reaction of 4-fluoro­aniline, ethyl aceto­acetate and benzaldehyde at room temperature using sodium lauryl sulfate as a catalyst. The com­pound crystallizes with two mol­ecules in the asymmetric unit. The tetra­hydro­pyridine ring adopts a distorted boat conformation in both mol­ecules and the dihedral angles between the planes of the fluoro-substituted rings are 77.1 (6) and 77.3 (6)°. The amino group and carbonyl O atom are involved in an intra­molecular N—H⋯O hydrogen bond, thereby generating an *S*(6) ring motif. In the crystal, mol­ecules are linked by C—H⋯F hydrogen bonds forming a three-dimensional network and C—H⋯π inter­actions. A Hirshfeld surface analysis of the crystal structure indicates that the most important contributions to the crystal packing are from H⋯H (47.9%), C⋯H/H⋯C (30.7%) and F⋯H/H⋯F (12.4%) contacts. The optimized structure calculated using density functional theory (DFT) at the B3LYP/6-311+G(2d,p) level is compared with the experimentally determined molecular structure in the solid state. The HOMO–LUMO behaviour was used to determine the energy gap and the Natural Bond Orbital (NBO) analysis was done to study donor–acceptor interconnections.

## Abstract 2

The one-pot synthesis, crystal structure, Hirshfeld surface analysis, DFT and NBO study of a highly functionalized tetra­hydro­pyridine are reported.

## 1. Chemical context

Highly functionalized tetra­hydro­pyridines are widely present in naturally occurring and synthetic drugs (Watson *et al.*, 2000 ▸), which exhibit many desirable pharmacological activities, such as hyperglycemic (Yeung *et al.*, 1982 ▸), analgesic (Rao *et al.*, 1995 ▸; Gangapuram *et al.*, 2006 ▸), anti­malarial (Misra *et al.*, 2009 ▸), nicotinic (Olesen *et al.*, 1998 ▸), anti-influenza (Chand *et al.*, 2001 ▸) and anti­convulsant properties (Ho *et al.*, 2001 ▸). Earlier literature shows that a lot of effort was devoted to develop a simple and easy protocol for the synthesis of substituted tetra­hydro­pyridines using various catalytic systems, such as bromo­dimethyl­sulfonium bromide (BDMS) (Khan *et al.*, 2008 ▸), iodine, tetra­butyl­ammonium tribromide (TBATB) (Khan *et al.*, 2010 ▸), cerium ammonium nitrate (Wang *et al.*, 2010 ▸), BF_3_·SiO_2_ (Ramachandran *et al.*, 2012 ▸), ZrOCl_2_·8H_2_O (Mishra & Ghosh, 2011 ▸), Bi(NO_3_)_3_·5H_2_O (Brahmchari & Das, 2012 ▸), oxalic acid (Sajadikhah *et al.*, 2012 ▸), picric acid (Mukhopadhyay *et al.*, 2011 ▸), AcOH (Lashkari *et al.*, 2013 ▸), l-proline/TFA (Misra *et al.*, 2009 ▸), InCl_3_ (Clarke *et al.*, 2008 ▸), zirconia pillared clay–polyphospho­ric acid (Kar *et al.*, 2014 ▸), silica sulfuric acid (Daraei *et al.*, 2015 ▸), graphene oxide (Gupta *et al.*, 2017 ▸), cyanuric chloride (Ramesh *et al.*, 2017 ▸), aluminized polyborate (Mali *et al.*, 2018 ▸) and thi­amine hydro­chloride (Singh *et al.*, 2020 ▸). These methodologies suffer from one or other disadvantages, such as a multi-step synthetic sequence, the requirement for expensive reagents or catalysts, *etc*. 
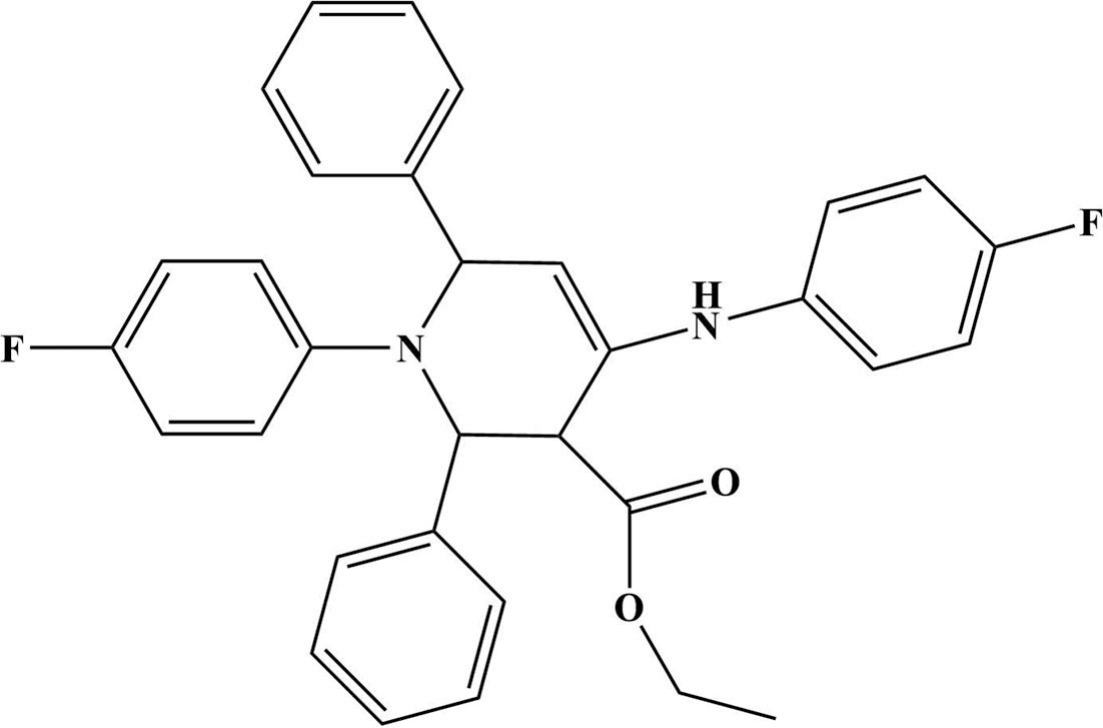


The development of improved synthetic procedures with an objective of green chemistry and technology, and the use of recyclable catalysts for organic synthesis to maximize efficiency and minimize waste, has been currently in demand. To accom­plish this objective, our laboratory has developed an ecofriendly catalyst for organic transformations; herein, this article describes the application of sodium lauryl sulfate (SLS) as an efficient and ecofriendly catalyst for tetra­hydro­pyridine synthesis in water at room temperature by the reaction of benzaldehyde, 4-fluoro­aniline and β-ketoester. This catalyst is environmentally benign due to its reusability and nontoxic nature; it is readily available and inexpensive, and this reaction can be regarded as an efficient approach for the preparation of synthetically and pharmaceutically important functionalized tetra­hydro­pyridine systems. To the best of our knowledge, this is the second report on the use of SLS for the synthesis of a highly functionalized tetra­hydro­pyridine (Bansal *et al.*, 2017 ▸). Herein, we report the synthesis, crystal structure and Hirshfeld surface analysis of ethyl 1-(4-fluoro­phen­yl)-4-[(4-fluoro­phen­yl)amino]-2,6-diphenyl-1,2,5,6-tetra­hydro­pyridine-3-carboxyl­ate, (**I**), using sodium lauryl sulfate as catalyst.

## 2. Structural commentary

**Figure 1:**
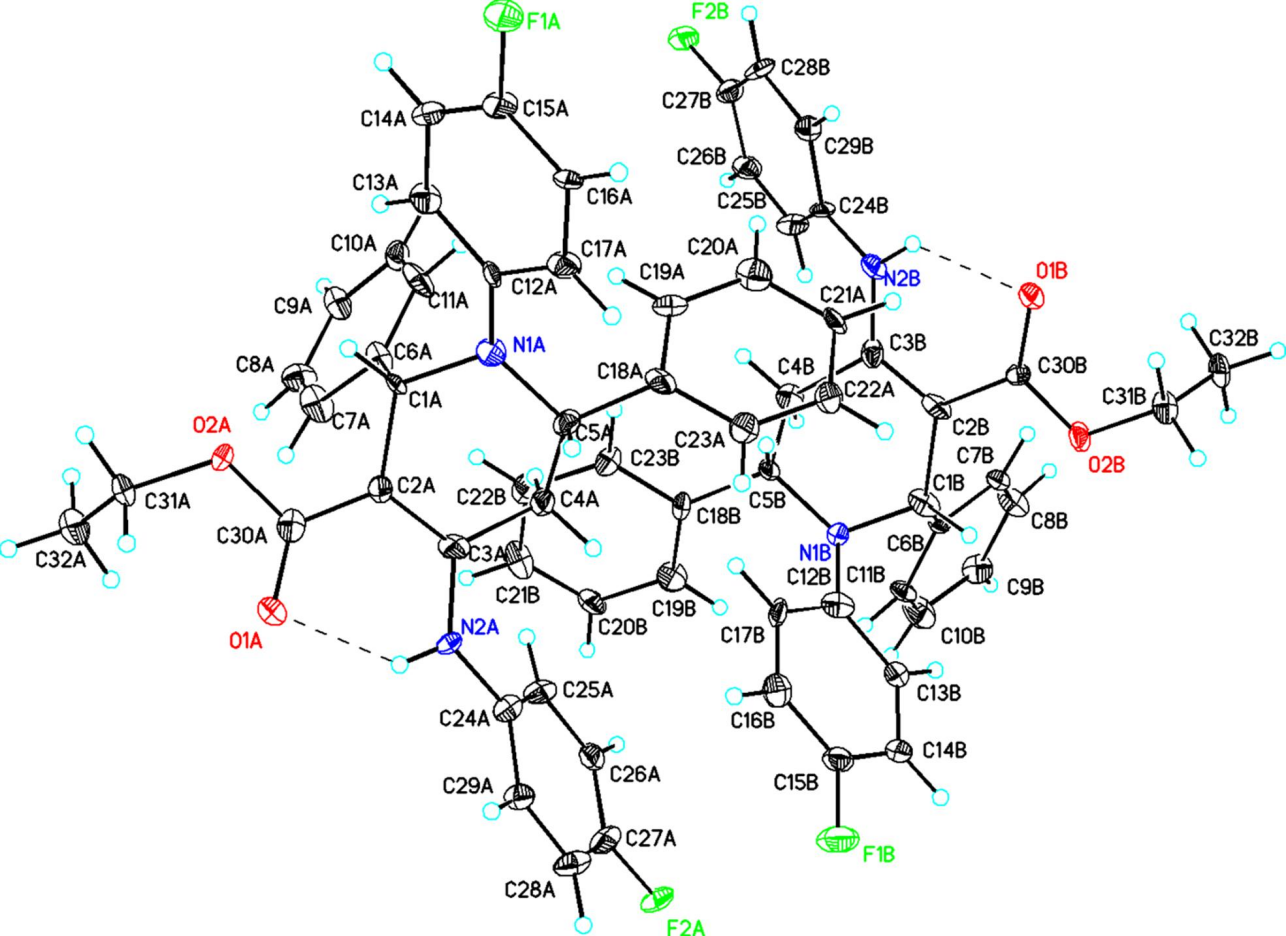
The asymmetric unit of (**I**), with displacement ellipsoids drawn at the 50% probability level. The N—H⋯O hydrogen bonds are depicted by dashed lines.

The title com­pound, (**I**) (Fig. 1 ▸), which is a rare example of fluoro­phenyl groups attached to the N atom of a central tetra­hydro­pyridine ring, crystallizes in a noncentrosymmetric space group (monoclinic, *P*2_1_). There are two mol­ecules in the asymmetric unit (*Z* = 4). In the arbitrarily chosen asymmetric unit, the stereogenic atoms C1*A*, C5*A*, C1*B* and C5*B* all have an *S* configuration. The absolute structure is not well established, but the racemic mol­ecule presumably spontaneously resolves into its enanti­omers upon crystallization. The tetra­hydro­pyridine ring adopts a distorted boat conformation in both mol­ecules. The fluoro­phenyl groups are attached to the tetra­hydro­pyridine ring in a pseudo-*para* orientation. The C—N—C—C torsion angles are 171.8 (10) and 161.0 (11)° in mol­ecule *A* (containing C1*A*), and 172.2 (9) and 160.9 (12)° in mol­ecule *B* containing C1*B*. The dihedral angles between the planes of the C12*A*–C17*A*/C18*A*–C23*A* and C12*B*–C17*B*/C18*B*–C23*B* rings are 77.1 (6) and 77.3 (6)°, respectively. The mean plane of the central tetra­hydro­pyridine N1*A*/C1*A*–C5*A* ring subtends dihedral angles of 74.0 (6), 45.9 (6), 46.4 (6) and 70.4 (6)° with the pendant phenyl C6*A*–C11*A*, C12*A*–C17*A*, C18*A*–C23*A* and C24*A*–C29*A* rings, respectively. Equivalent data for the N1*B*/C1*B*–C5*B* ring and the C6*B*–C11*B*, C12*B*–C17*B*, C18*B*–C23*B* and C24*B*–C29*B* phenyl groups are 76.2 (6), 48.7 (6), 45.0 (6), 71.5 (6)°, respectively. In both mol­ecules, the amine N atoms are clearly nonplanar, with the sum of the bond angles around N1*A* and N2*A* being 351.0 and 359.0°, respectively, and those around N1*B* and N2*B* being 351.4 and 347.3°, respectively. Otherwise, all bond lengths and angles are com­parable to those observed in related structures (Anthal *et al.*, 2013*a* ▸; Yu *et al.*, 2013 ▸). In both mol­ecules, the amine N atom participates in an intra­molecular N—H⋯O hydrogen bond of length *ca* 2.65 Å with the O1 atom of the carbonyl group, thereby generating an *S*(6) ring, essentially similar to those in [Ph(C_6_H_4_N)Ph(NH)(FC_6_H_4_)_2_(OCOC_2_H_5_)] [2.672 (3) Å; Anthal *et al.*, 2013*a* ▸] and [Ph(C_6_H_4_N)Ph(NH)(ClC_6_H_4_)_2_(OCOC_2_H_5_)] [2.659 (5) Å; Yu *et al.*, 2013 ▸].

## 3. Supra­molecular features

**Figure 2:**
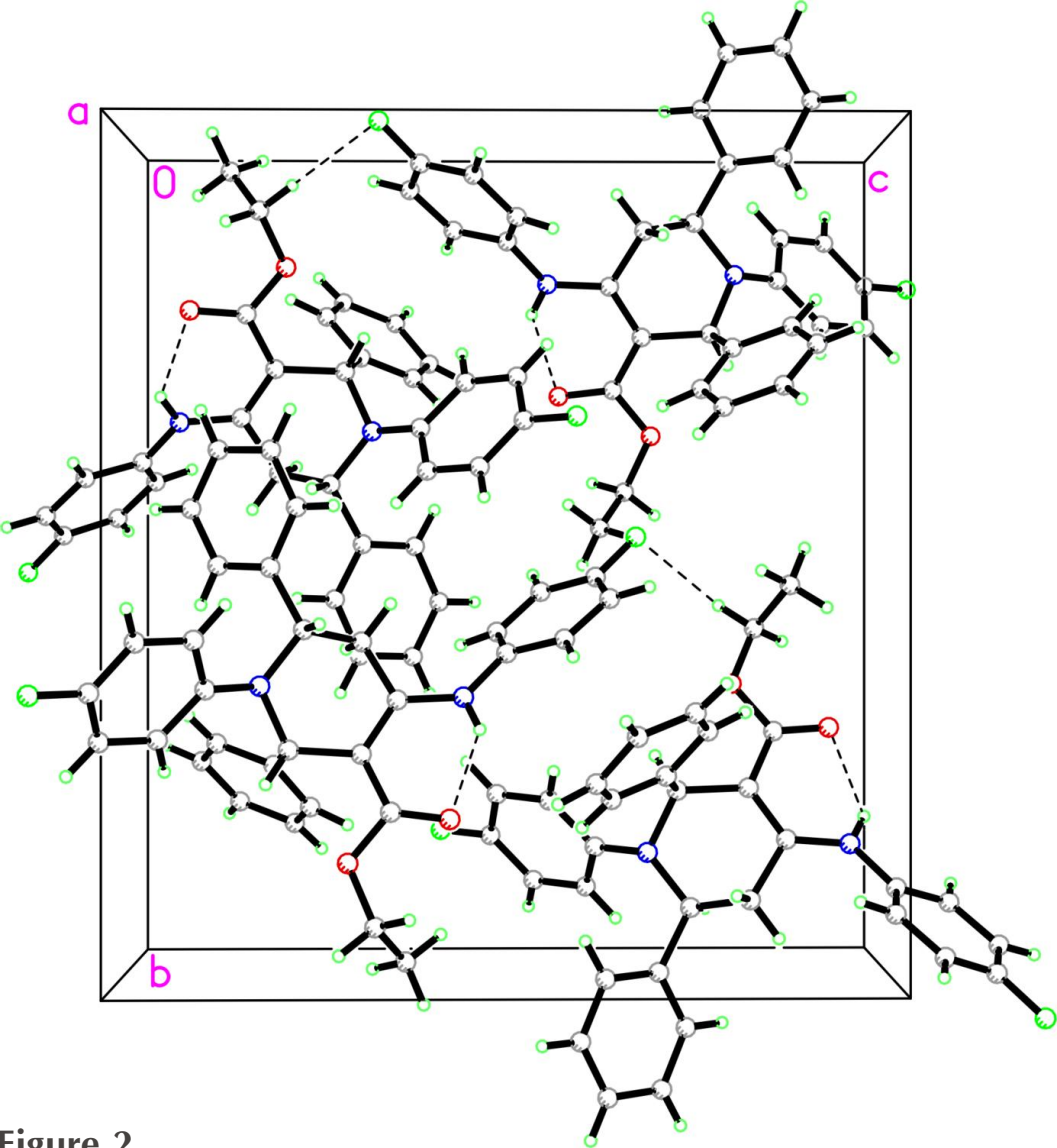
Packing diagram of (**I**), viewed along the *a* axis. Dashed lines indicate N—H⋯O hydrogen bonds and inter­molecular C—H⋯F inter­actions.

The crystal packing of (**I**), viewed along the *a* axis, is presented in Fig. 2 ▸. The com­pound packs in a way that allows close contacts between the F and H atoms of adjacent mol­ecules, leading to a network of C—H⋯F inter­actions (Table 1 ▸). Furthermore, there are six C—H⋯π inter­actions (Table 1 ▸), which may help to consolidate the packing.

## 4. Hirshfeld surface analysis and com­putational chemistry

**Figure 3:**
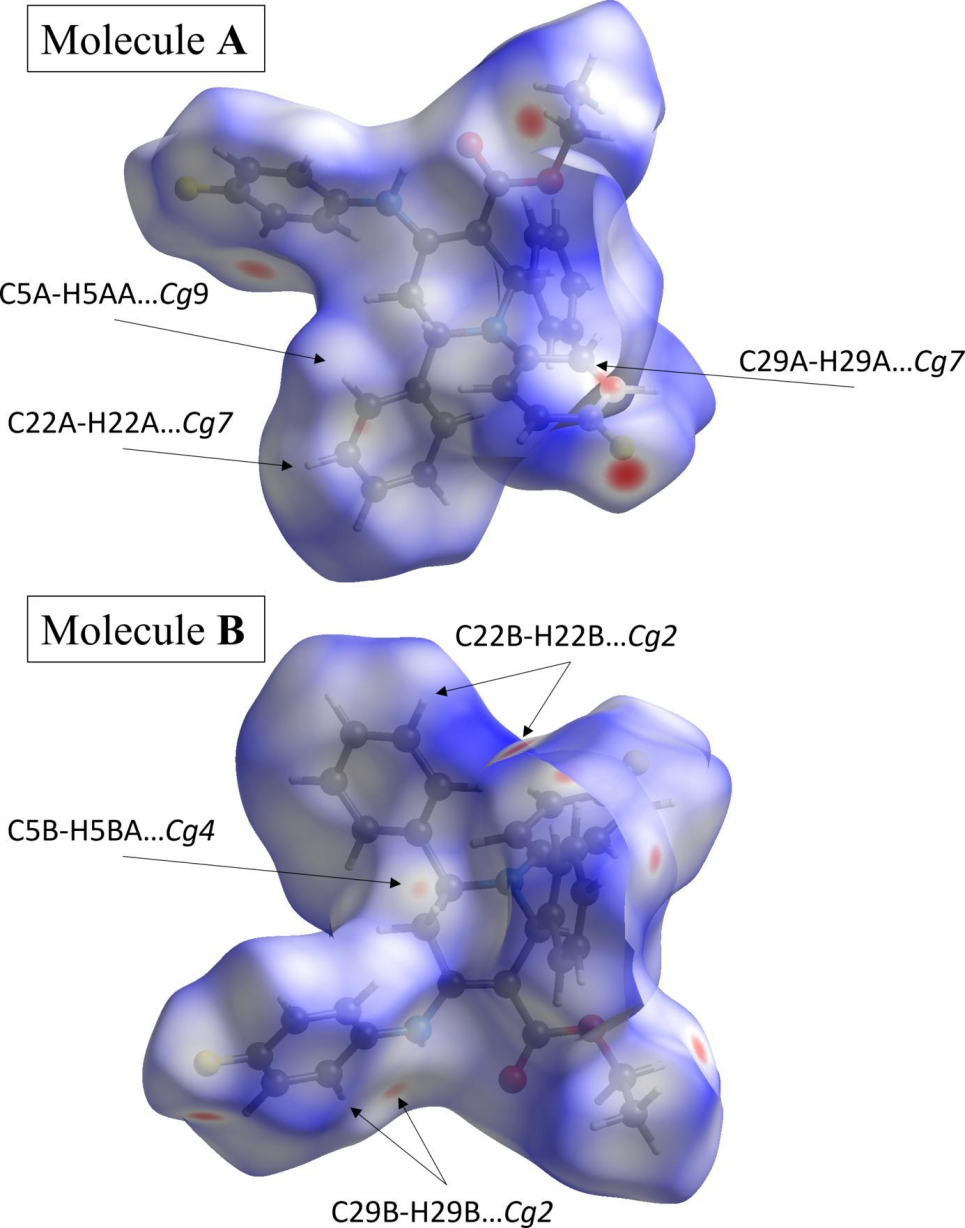
A view of the three-dimensional Hirshfeld surface mapped over *d*_norm_ in the range from −0.25 to 1.48 a.u. for mol­ecule *A* and from −0.25 to 1.43 a.u. for mol­ecule *B*.

The Hirshfeld surface analysis was performed with *CrystalExplorer* (Version 21.5; Spackman *et al.*, 2021 ▸). Fig. 3 ▸ shows views of the *d*_norm_ surfaces for the two mol­ecules in the asym­metric unit plotted over the limits from −0.25 to 1.48 a.u. for mol­ecule **1** and −0.25 to 1.43 a.u. for mol­ecule **2**. The red spots that appear around atoms F1 and F2 in mol­ecules *A* and *B* are caused by inter­molecular C31*A*—H31*A*⋯F2*B*, C31*A*—H31*B*⋯F1*B*, C31*B*—H31*C*⋯F2*A* and C31*B*—H31*D*⋯F1*A* inter­actions (Table 2 ▸). An intra­molecular N—H⋯O hydrogen bond is also indicated by the red spots near the H and O atoms [Figs. 3 ▸(*a*) and 3(*b*)].

**Figure 4:**
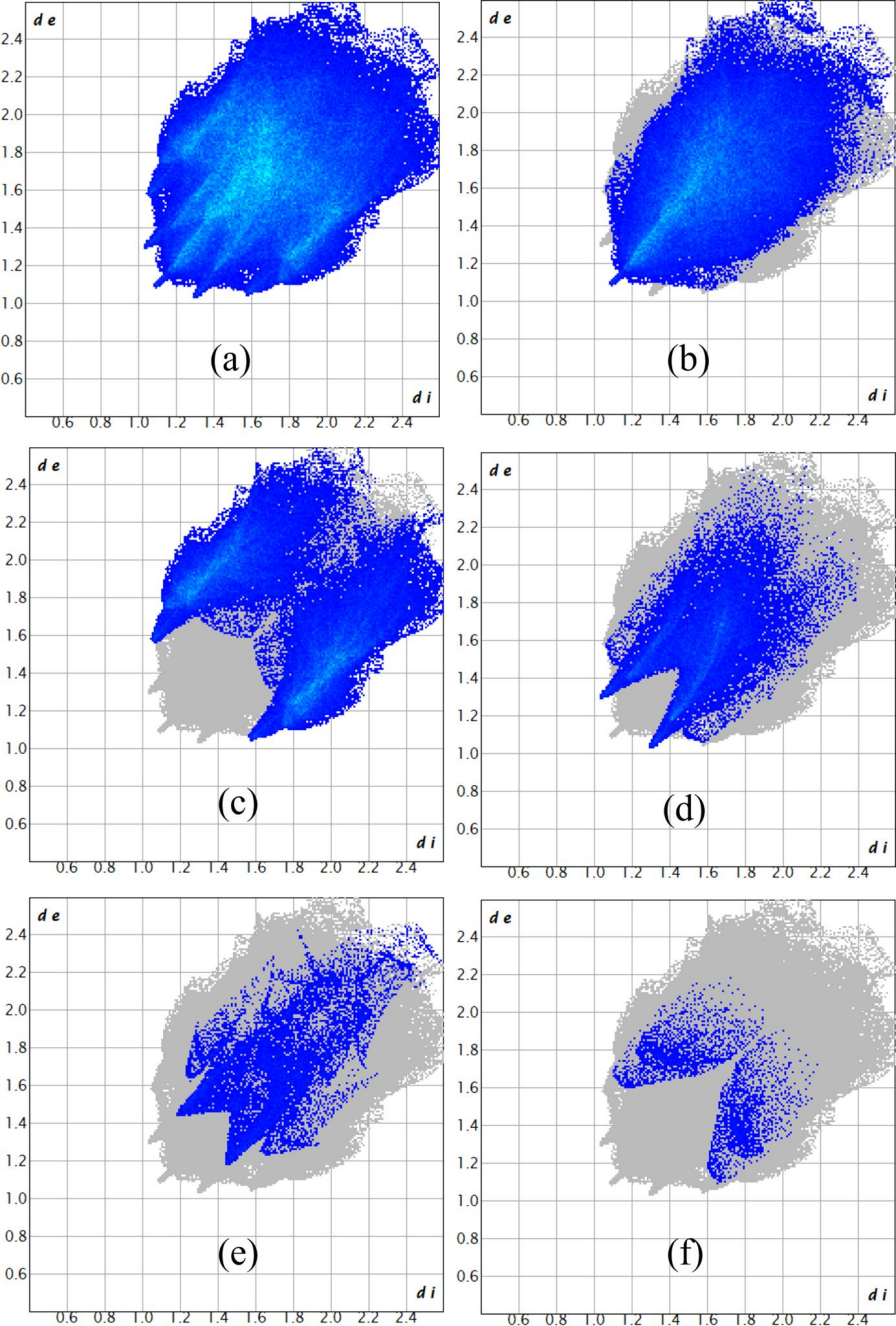
A view of the two-dimensional fingerprint plots for the title com­pound, showing (*a*) all inter­actions, and those delineated into (*b*) H⋯H, (*c*) C⋯H/H⋯C, (*d*) F⋯H/H⋯F, (*e*) O⋯H/H⋯O and (*f*) N⋯H/H⋯N inter­actions. The *d*_i_ and *d*_e_ values are the closest inter­nal and external distances (in Å) from given points on the Hirshfeld surface.

The two-dimensional fingerprint plots were generated using *CrystalExplorer* encom­passing all inter­molecular contacts, as well as the delineated specific contacts (Fig. 4 ▸). The most significant contacts and their percentage contributions to the Hirshfeld surface are given in Table 2 ▸. The most important inter­action is H⋯H, contributing 47.9% to the crystal packing. The presence of C—H⋯F inter­actions is indicated by pairs of characteristic wings in the fingerprint plot representing C⋯H/H⋯C and F⋯H/H⋯F contacts, with contributions of 30.7 and 12.4%, respectively, to the HS. The lowest contributions are from O⋯H/H⋯O (4.9%), N⋯H/H⋯N (1.3%) and F⋯C/C⋯F (0.8%) contacts.

**Figure 5:**
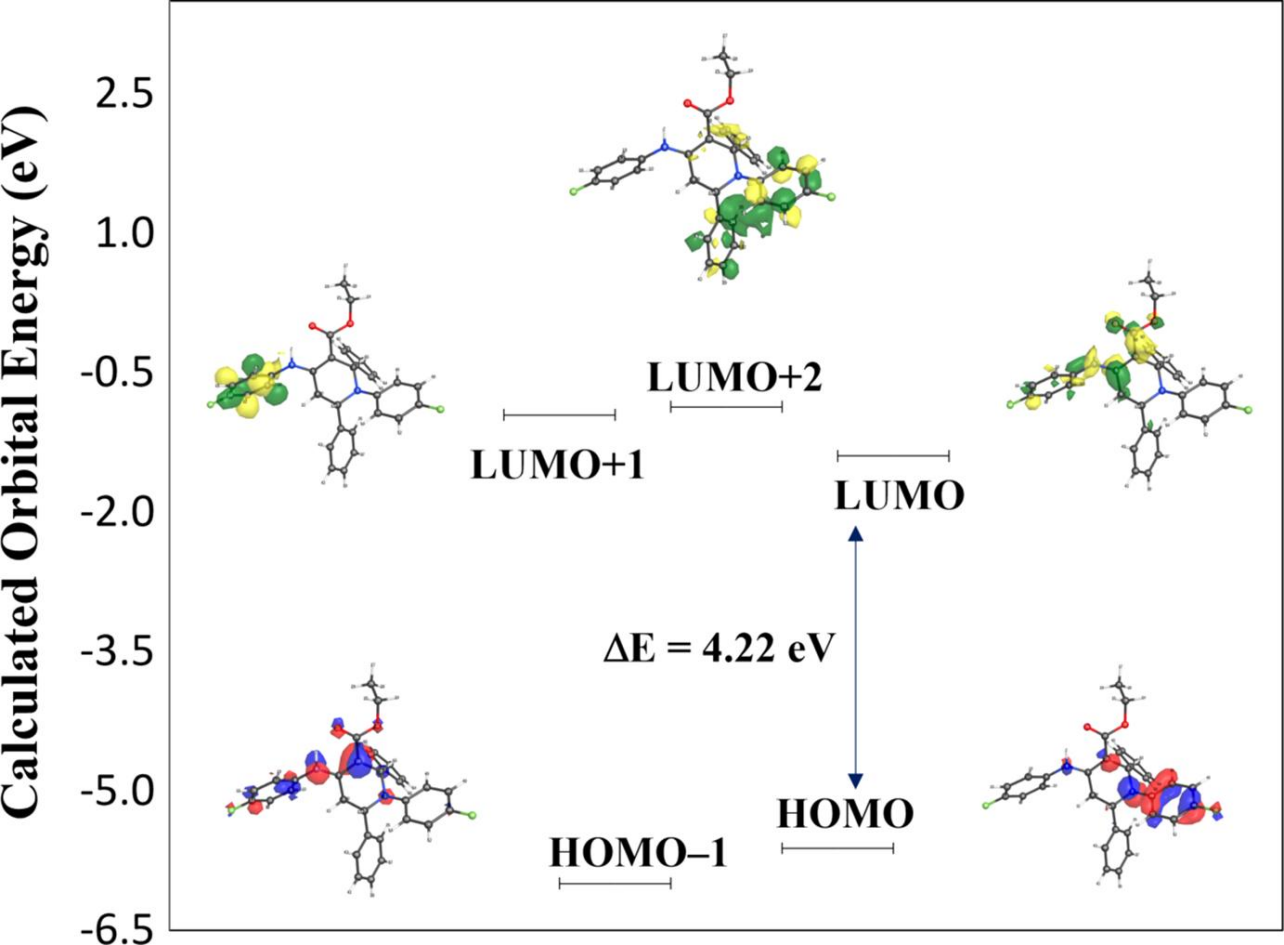
HOMO–LUMO energy diagram for the title com­pound.

A density functional theory (DFT) geometry-optimized mol­ecular orbital calculation (*WebMOPro*; Polik & Schmidt, 2021 ▸) with the *GAUSSIAN16* program package employing the B3LYP functional and 6-311+G(2d,p) basis set (Becke, 1993 ▸) was performed on (**I**) with the starting geometries taken from the X-ray refinement data. The theoretical and experimental results related to bond lengths and angles are in good agreement (see Table S1 in the supporting information) and calculated numerical values are collated in Table S2. The calculated HOMO–LUMO energy gap is 4.22 eV (Fig. 5 ▸). An NBO analysis was performed on (**I**) at the DFT level using the B3LYP method and 6-311+G(2d,p) basis set. The perturbation energies of the donor–acceptor inter­actions are tabulated in Table S3.

## 5. Database survey

A search of the Cambridge Structural Database (CSD, Version 5.44, update April 2023; Groom *et al.*, 2016 ▸) for the basic skeleton of this com­pound gave 50 hits. Most of these contain the search fragment as part of a larger mol­ecule, but three are considered similar to the title com­pound. These are ethyl 4-anilino-2,6-bis­(4-fluoro­phen­yl)-1-phenyl-1,2,5,6-tetra­hydro­pyridine-3-carboxyl­ate (CSD refcode LETBET; Anthal *et al.*, 2013*a* ▸), in which the central tetra­hydro­pyridine ring unit is similar to that in (**I**), *anti*-ethyl 4-anilino-1,2,6-triphenyl-1,2,5,6-tetra­hydro­pyridine-3-carboxyl­ate (VOLDIK; Khan *et al.*, 2008 ▸), in which the 2- and 6-positions of the piperidine was shown to be *anti*, and ethyl 2,6-bis­(4-chloro­phen­yl)-1-(4-fluoro­phen­yl)-4-[(4-fluoro­phen­yl)amino]-1,2,5,6-tetra­hydro­pyridine-3-carboxyl­ate (WIHCOH; Anthal *et al.*, 2013*b* ▸), in which the tetra­hydro­pyridine unit is similar to that in (**I**).

## 6. Synthesis and crystallization

The title com­pound was obtained by the one-pot multi-com­ponent reaction using sodium lauryl sulfate (SLS) as catalyst. In a typical experiment, a mixture of 4-fluoro­aniline (2 mmol) and ethyl aceto­acetate (1 mmol) in 10 ml water was stirred for 10 min in the presence of 0.02 g SLS at room temperature. To this solution was added benzaldehyde (2 mmol) and the reaction mixture was stirred for 30 min. The progress of reactions was monitored by thin-layer chromatography (TLC), eluted with an ethyl acetate and *n*-hexane (3:7 *v*/*v*) mixture. After com­pletion of the reaction, a thick precipitate was filtered off and washed with water. Colourless plate-shaped crystals suitable for X-ray diffraction analysis were obtained by slow evaporation from ethanol solution.

Yield 81%, m.p. 443 K. FT–IR (selected): (ν, cm^−1^): 3246, 3190, 3080, 2974, 1680, 1645, 1604, 1585, 1492, 1450, 1249, 1072, 941, 802, 698. ^1^H NMR [400 MHz, CDCl_3_, δ (ppm)]: 10.26 (*br s*, 1H), 7.31–7.27 (*m*, 8H), 7.19–7.17 (*d*, *J* = 8.0 Hz, 1H), 7.09–7.07 (*d*, *J* = 8.0 Hz, 2H), 7.04–7.02 (*d*, *J* = 8.2 Hz, 2H), 6.48–6.46 (*d*, *J* = 8.0 Hz, 2H), 6.43 (*s*, 1H), 6.21–6.19 (*d*, *J* = 8.0 Hz, 2H), 5.14–5.13 (*s*, 1H), 4.50–4.46 (*d*, *J* = 16.0 Hz, 2H), 4.38–4.35 (*q*, *J* = 12.0 Hz, 2H), 2.75–2.72 (*t*, *J* = 24.0 Hz, 1H), 1.52–1.49 (*t*, *J* = 12.0 Hz, 3H). ^13^C NMR (100 MHz, CDCl_3_, ppm): 14.8, 33.5, 55.3, 58.3, 114.0, 121.2, 126.3, 126.5, 126.6, 127.0, 127.5, 128.4, 128.7, 128.8, 129.0, 131.4, 136.4, 142.3, 143.3, 145.5, 155.4, 168.1.

## 7. Refinement details

Crystal data, data collection and structure refinement details are summarized in Table 3 ▸. H atoms attached to carbon were placed in calculated positions (C—H = 0.95–1.00 Å), while those attached to nitro­gen were placed in locations derived from a difference map and their coordinates were adjusted to give N—H = 0.85 Å. All were included as riding contributions with isotropic displacement parameters 1.2–1.5 times those of the attached atoms.

## Supplementary Material

Crystal structure: contains datablock(s) I. DOI: 10.1107/S205698902300748X/hb8074sup1.cif

Structure factors: contains datablock(s) I. DOI: 10.1107/S205698902300748X/hb8074Isup2.hkl

Supporting information file. DOI: 10.1107/S205698902300748X/hb8074Isup3.cml

CCDC reference: 2290952

Additional supporting information:  crystallographic information; 3D view; checkCIF report

## Figures and Tables

**Table 1:** Hydrogen-bond geometry (Å, °) *Cg*2, *Cg*4, *Cg*7 and *Cg*9 are the centroids of the C6*A*–C11*A*, C18*A*–C23*A*, C6*B*–C11*B* and C18*B*–C23*B* rings, respectively.

*D*—H⋯*A*	*D*—H	H⋯*A*	*D*⋯*A*	*D*—H⋯*A*
N2*A*—H2*AA*⋯O1*A*	0.85 (3)	1.98 (10)	2.652 (13)	136 (12)
N2*B*—H2*BA*⋯O1*B*	0.88	2.04	2.664 (13)	127
C31*A*—H31*A*⋯F2*B*	0.99	2.43	3.327 (14)	151
C31*A*—H31*B*⋯F1*B*	0.99	2.40	3.257 (15)	144
C31*B*—H31*C*⋯F2*A*	0.99	2.45	3.213 (15)	134
C31*B*—H31*D*⋯F1*A*	0.99	2.32	3.281 (13)	165
C5*A*—H5*AA*⋯*Cg*9^i^	1.00	2.91	3.907 (13)	178
C5*B*—H5*BA*⋯*Cg*4^ii^	1.00	2.96	3.958 (14)	174
C22*A*—H22*A*⋯*Cg*7^i^	0.95	2.87	3.770 (14)	159
C22*B*—H22*B*⋯*Cg*2^ii^	0.95	2.94	3.857 (15)	162
C29*A*—H29*A*⋯*Cg*7^iii^	0.95	2.71	3.437 (13)	134
C29*B*—H29*B*⋯*Cg*2^iv^	0.95	2.84	3.595 (13)	138

Symmetry codes: (i) 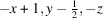 ; (ii) 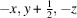 ; (iii) 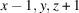 ; (iv) 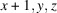 .

**Table 2:** Percentage contributions of inter­atomic contacts to the Hirshfeld surface for the title com­pound

Contact	Percentage contribution
H⋯H	47.9
C⋯H/H⋯C	30.7
F⋯H/H⋯F	12.4
O⋯H/H⋯O	4.9
N⋯H/H⋯N	1.3
F⋯C/C⋯F	0.8
C⋯C	0.7
C⋯O/O⋯C	0.6
F⋯F	0.5
F⋯O/O⋯F	0.2

**Table 3:** Experimental details

Crystal data	
Chemical formula	C_32_H_28_F_2_N_2_O_2_
*M*_r_	510.56
Crystal system, space group	Monoclinic, *P*2_1_
Temperature (K)	100
*a*, *b*, *c* (Å)	8.8072 (12), 17.795 (2), 16.222 (2)
β (°)	91.317 (9)
*V* (Å^3^)	2541.7 (6)
*Z*	4
Radiation type	Mo *K*α
μ (mm^−1^)	0.09
Crystal size (mm)	0.31 × 0.24 × 0.09

Data collection	
Diffractometer	Bruker APEXII CCD
Absorption correction	Multi-scan (*SADABS*; Sheldrick, 1996 ▸)
*T*_min_, *T*_max_	0.544, 0.745
No. of measured, independent and observed [*I* > 2σ(*I*)] reflections	44124, 10842, 8132
*R*_int_	0.153
(sin θ/λ)_max_ (Å^−1^)	0.636

Refinement	
*R*[*F* ^2^ > 2σ(*F* ^2^)], *wR*(*F* ^2^), *S*	0.118, 0.324, 1.17
No. of reflections	10842
No. of parameters	689
No. of restraints	75
H-atom treatment	H atoms treated by a mixture of independent and constrained refinement
Δρ_max_, Δρ_min_ (e Å^−3^)	1.04, −0.54
Absolute structure	Flack *x* determined using 2653 quotients [(*I* ^+^) − (*I* ^−^)]/[(*I* ^+^) + (*I* ^−^)] (Parsons *et al.*, 2013 ▸)
Absolute structure parameter	−0.4 (7)

Computer programs: *APEX2* (Bruker, 2005 ▸), *SAINT* (Bruker, 2005 ▸), *SHELXT* (Sheldrick, 2015*a* ▸), *SHELXL2019* (Sheldrick, 2015*b* ▸), *SHELXTL* (Sheldrick, 2008 ▸) and *publCIF* (Westrip, 2010 ▸).

## supplementary crystallographic information

### Crystal data

**Table:**

C_32_H_28_F_2_N_2_O_2_	*F*(000) = 1072
*M**_r_* = 510.56	*D*_x_ = 1.334 Mg m^−^^3^
Monoclinic, *P*2_1_	Mo *K*α radiation, λ = 0.71073 Å
*a* = 8.8072 (12) Å	Cell parameters from 8110 reflections
*b* = 17.795 (2) Å	θ = 2.5–25.9°
*c* = 16.222 (2) Å	µ = 0.09 mm^−^^1^
β = 91.317 (9)°	*T* = 100 K
*V* = 2541.7 (6) Å^3^	Plate, colorless
*Z* = 4	0.31 × 0.24 × 0.09 mm

### Data collection

**Table:**

Bruker APEX-II CCD diffractometer	8132 reflections with *I* > 2σ(*I*)
ω scans	*R*_int_ = 0.153
Absorption correction: multi-scan (SADABS; Sheldrick, 1996)	θ_max_ = 26.9°, θ_min_ = 1.7°
*T*_min_ = 0.544, *T*_max_ = 0.745	*h* = −11→11
44124 measured reflections	*k* = −22→22
10842 independent reflections	*l* = −20→20

### Refinement

**Table:**

Refinement on *F*^2^	Secondary atom site location: difference Fourier map
Least-squares matrix: full	Hydrogen site location: mixed
*R*[*F*^2^ > 2σ(*F*^2^)] = 0.118	H atoms treated by a mixture of independent and constrained refinement
*wR*(*F*^2^) = 0.324	*w* = 1/[σ^2^(*F*_o_^2^) + (0.2*P*)^2^] where *P* = (*F*_o_^2^ + 2*F*_c_^2^)/3
*S* = 1.17	(Δ/σ)_max_ = 0.001
10842 reflections	Δρ_max_ = 1.04 e Å^−^^3^
689 parameters	Δρ_min_ = −0.54 e Å^−^^3^
75 restraints	Absolute structure: Flack *x* determined using 2653 quotients [(I+)-(I-)]/[(I+)+(I-)] (Parsons *et al.*, 2013)
Primary atom site location: dual	Absolute structure parameter: −0.4 (7)

### Special details

**Table:**

Geometry. All e.s.d.'s (except the e.s.d. in the dihedral angle between two l.s. planes) are estimated using the full covariance matrix. The cell e.s.d.'s are taken into account individually in the estimation of e.s.d.'s in distances, angles and torsion angles; correlations between e.s.d.'s in cell parameters are only used when they are defined by crystal symmetry. An approximate (isotropic) treatment of cell e.s.d.'s is used for estimating e.s.d.'s involving l.s. planes.
Refinement. Refined as a 2-component twin.

### Fractional atomic coordinates and isotropic or equivalent isotropic displacement parameters (Å^2^)

**Table:**

	*x*	*y*	*z*	*U*_iso_*/*U*_eq_	
F1A	1.2145 (9)	0.6537 (4)	−0.0730 (4)	0.0282 (18)	
F2A	0.5317 (9)	0.4785 (4)	0.6692 (4)	0.0288 (18)	
O1A	0.9193 (11)	0.8004 (4)	0.4287 (5)	0.0213 (18)	
O2A	0.9079 (10)	0.8507 (4)	0.3013 (5)	0.0202 (19)	
N1A	0.8252 (12)	0.6508 (6)	0.1882 (6)	0.019 (2)	
N2A	0.8030 (13)	0.6640 (5)	0.4451 (6)	0.021 (2)	
H2AA	0.854 (14)	0.700 (5)	0.466 (8)	0.025*	
C1A	0.7854 (14)	0.7253 (6)	0.2229 (7)	0.017 (2)	
H1AA	0.853893	0.762724	0.196791	0.020*	
C2A	0.8189 (14)	0.7291 (6)	0.3160 (7)	0.017 (2)	
C3A	0.7776 (13)	0.6676 (7)	0.3607 (7)	0.016 (2)	
C4A	0.7204 (14)	0.6012 (6)	0.3143 (7)	0.018 (2)	
H4AA	0.616757	0.611138	0.291943	0.021*	
H4AB	0.716095	0.556989	0.351191	0.021*	
C5A	0.8287 (14)	0.5858 (6)	0.2437 (7)	0.016 (2)	
H5AA	0.934099	0.578633	0.266529	0.019*	
C6A	0.6241 (13)	0.7476 (6)	0.1984 (7)	0.017 (2)	
C7A	0.5398 (14)	0.8000 (7)	0.2428 (8)	0.024 (3)	
H7AA	0.582800	0.819602	0.292566	0.028*	
C8A	0.3983 (14)	0.8244 (7)	0.2180 (8)	0.022 (3)	
H8AA	0.343692	0.857768	0.252109	0.027*	
C9A	0.3332 (17)	0.8006 (7)	0.1425 (8)	0.028 (3)	
H9AA	0.236874	0.818845	0.124048	0.033*	
C10A	0.4142 (14)	0.7492 (6)	0.0953 (8)	0.020 (2)	
H10A	0.371490	0.731408	0.044588	0.024*	
C11A	0.5557 (14)	0.7239 (7)	0.1215 (8)	0.023 (3)	
H11A	0.609210	0.689818	0.087717	0.028*	
C12A	0.9314 (13)	0.6528 (6)	0.1248 (6)	0.013 (2)	
C13A	0.9190 (13)	0.7101 (7)	0.0646 (7)	0.018 (2)	
H13A	0.844532	0.748345	0.069360	0.021*	
C14A	1.0168 (15)	0.7099 (7)	−0.0016 (8)	0.021 (3)	
H14A	1.011386	0.748455	−0.042093	0.026*	
C15A	1.1210 (14)	0.6530 (7)	−0.0073 (7)	0.020 (2)	
C16A	1.1361 (15)	0.5967 (7)	0.0496 (7)	0.021 (3)	
H16A	1.211869	0.559163	0.044266	0.025*	
C17A	1.0379 (13)	0.5957 (7)	0.1156 (7)	0.018 (2)	
H17A	1.043535	0.555931	0.154614	0.021*	
C18A	0.7769 (13)	0.5144 (7)	0.1994 (7)	0.017 (2)	
C19A	0.6800 (14)	0.5125 (7)	0.1287 (7)	0.019 (2)	
H19A	0.646179	0.558704	0.105421	0.023*	
C20A	0.6324 (14)	0.4458 (8)	0.0921 (8)	0.025 (3)	
H20A	0.570601	0.446242	0.043302	0.029*	
C21A	0.6779 (17)	0.3771 (7)	0.1290 (7)	0.025 (3)	
H21A	0.646713	0.330565	0.105462	0.029*	
C22A	0.7687 (16)	0.3787 (7)	0.2000 (8)	0.025 (3)	
H22A	0.797499	0.332583	0.225436	0.030*	
C23A	0.8181 (15)	0.4449 (7)	0.2344 (7)	0.021 (3)	
H23A	0.881145	0.443783	0.282642	0.025*	
C24A	0.7268 (13)	0.6141 (7)	0.4997 (7)	0.015 (2)	
C25A	0.5808 (13)	0.5914 (7)	0.4869 (7)	0.017 (2)	
H25A	0.524947	0.607559	0.439395	0.021*	
C26A	0.5136 (15)	0.5440 (7)	0.5442 (7)	0.019 (2)	
H26A	0.414461	0.524402	0.534433	0.023*	
C27A	0.5950 (14)	0.5262 (7)	0.6154 (7)	0.019 (3)	
C28A	0.7379 (13)	0.5508 (7)	0.6313 (7)	0.020 (2)	
H28A	0.789702	0.537041	0.681068	0.024*	
C29A	0.8093 (15)	0.5970 (7)	0.5731 (7)	0.021 (3)	
H29A	0.908936	0.615946	0.582808	0.025*	
C30A	0.8857 (13)	0.7933 (6)	0.3544 (7)	0.017 (2)	
C31A	0.9685 (15)	0.9203 (6)	0.3367 (8)	0.020 (2)	
H31A	1.019282	0.949562	0.293281	0.024*	
H31B	1.045222	0.908244	0.380241	0.024*	
C32A	0.8443 (17)	0.9665 (7)	0.3726 (8)	0.029 (3)	
H32A	0.887315	1.012885	0.395932	0.044*	
H32B	0.769217	0.979120	0.329256	0.044*	
H32C	0.795121	0.937857	0.416099	0.044*	
F1B	0.7271 (9)	0.3382 (5)	0.5895 (4)	0.0304 (18)	
F2B	−0.0100 (10)	0.5223 (4)	−0.1668 (4)	0.0329 (19)	
O1B	0.4225 (11)	0.2050 (5)	0.0798 (5)	0.0229 (19)	
O2B	0.4196 (10)	0.1528 (4)	0.2072 (5)	0.0198 (18)	
N1B	0.3238 (11)	0.3488 (5)	0.3190 (5)	0.0145 (19)	
N2B	0.2858 (12)	0.3375 (6)	0.0594 (6)	0.018 (2)	
H2BA	0.354557	0.308190	0.037569	0.022*	
C1B	0.2806 (12)	0.2748 (7)	0.2817 (7)	0.017 (2)	
H1BA	0.348035	0.236205	0.308500	0.021*	
C2B	0.3132 (13)	0.2727 (7)	0.1889 (7)	0.016 (2)	
C3B	0.2687 (13)	0.3315 (6)	0.1419 (7)	0.017 (2)	
C4B	0.2144 (13)	0.3993 (7)	0.1903 (7)	0.017 (2)	
H4BA	0.111102	0.389769	0.210747	0.021*	
H4BB	0.209976	0.444022	0.153993	0.021*	
C5B	0.3249 (15)	0.4137 (6)	0.2634 (7)	0.019 (3)	
H5BA	0.429634	0.420071	0.241909	0.022*	
C6B	0.1226 (13)	0.2530 (6)	0.2997 (6)	0.013 (2)	
C7B	0.0376 (14)	0.1990 (6)	0.2501 (7)	0.017 (2)	
H7BA	0.082555	0.179232	0.202045	0.020*	
C8B	−0.1027 (16)	0.1759 (8)	0.2695 (8)	0.026 (3)	
H8BA	−0.155672	0.142312	0.233550	0.031*	
C9B	−0.1726 (14)	0.2003 (7)	0.3418 (8)	0.022 (3)	
H9BA	−0.268637	0.181456	0.357452	0.026*	
C10B	−0.0921 (16)	0.2548 (8)	0.3905 (8)	0.030 (3)	
H10B	−0.137923	0.275706	0.437790	0.036*	
C11B	0.0476 (13)	0.2767 (7)	0.3700 (7)	0.015 (2)	
H11B	0.099352	0.310785	0.405830	0.018*	
C12B	0.4325 (12)	0.3468 (7)	0.3852 (7)	0.016 (2)	
C13B	0.4196 (16)	0.2909 (7)	0.4453 (7)	0.023 (3)	
H13B	0.341036	0.254565	0.439633	0.027*	
C14B	0.5194 (16)	0.2871 (7)	0.5134 (7)	0.023 (3)	
H14B	0.510508	0.248077	0.552897	0.028*	
C15B	0.6305 (14)	0.3409 (7)	0.5221 (7)	0.020 (2)	
C16B	0.6480 (14)	0.3964 (7)	0.4645 (7)	0.021 (2)	
H16B	0.727042	0.432396	0.470803	0.025*	
C17B	0.5467 (14)	0.3994 (6)	0.3957 (7)	0.017 (2)	
H17B	0.557315	0.438215	0.356067	0.020*	
C18B	0.2774 (14)	0.4867 (6)	0.3053 (7)	0.019 (3)	
C19B	0.1929 (14)	0.4892 (7)	0.3747 (8)	0.023 (3)	
H19B	0.162808	0.443980	0.400826	0.028*	
C20B	0.1509 (16)	0.5585 (7)	0.4072 (7)	0.026 (3)	
H20B	0.092136	0.559260	0.455561	0.031*	
C21B	0.1912 (13)	0.6262 (7)	0.3719 (8)	0.023 (3)	
H21B	0.158869	0.672692	0.394300	0.027*	
C22B	0.2821 (16)	0.6237 (8)	0.3014 (8)	0.027 (3)	
H22B	0.315915	0.668761	0.276449	0.033*	
C23B	0.3213 (13)	0.5542 (6)	0.2691 (7)	0.017 (2)	
H23B	0.380027	0.552553	0.220758	0.020*	
C24B	0.2059 (14)	0.3854 (6)	0.0048 (6)	0.016 (2)	
C25B	0.0596 (13)	0.4079 (7)	0.0145 (7)	0.017 (2)	
H25B	0.006401	0.391756	0.061630	0.020*	
C26B	−0.0124 (15)	0.4534 (7)	−0.0424 (7)	0.022 (3)	
H26B	−0.113603	0.469611	−0.033848	0.026*	
C27B	0.0630 (14)	0.4757 (7)	−0.1124 (7)	0.020 (2)	
C28B	0.2055 (13)	0.4519 (7)	−0.1262 (7)	0.018 (2)	
H28B	0.254965	0.466150	−0.175180	0.022*	
C29B	0.2809 (14)	0.4058 (6)	−0.0677 (7)	0.019 (2)	
H29B	0.381249	0.388659	−0.076974	0.023*	
C30B	0.3901 (13)	0.2085 (6)	0.1534 (7)	0.015 (2)	
C31B	0.4960 (13)	0.0880 (6)	0.1744 (8)	0.018 (2)	
H31C	0.549866	0.061068	0.219784	0.022*	
H31D	0.572414	0.104610	0.134489	0.022*	
C32B	0.3846 (19)	0.0354 (7)	0.1322 (8)	0.032 (3)	
H32D	0.439203	−0.008036	0.110512	0.048*	
H32E	0.309921	0.018318	0.171926	0.048*	
H32F	0.332443	0.061810	0.086729	0.048*	

### Atomic displacement parameters (Å^2^)

**Table:**

	*U*^11^	*U*^22^	*U*^33^	*U*^12^	*U*^13^	*U*^23^
F1A	0.035 (4)	0.028 (4)	0.022 (4)	0.003 (3)	0.015 (3)	0.000 (3)
F2A	0.049 (5)	0.023 (4)	0.015 (3)	−0.002 (3)	0.007 (3)	0.011 (3)
O1A	0.032 (5)	0.017 (4)	0.015 (4)	−0.001 (4)	0.001 (4)	−0.004 (3)
O2A	0.036 (5)	0.010 (4)	0.014 (4)	−0.005 (3)	0.003 (4)	0.002 (3)
N1A	0.023 (5)	0.018 (5)	0.017 (5)	0.005 (4)	0.011 (4)	−0.001 (4)
N2A	0.039 (6)	0.013 (5)	0.010 (4)	−0.006 (4)	0.006 (4)	0.004 (4)
C1A	0.032 (7)	0.010 (5)	0.009 (5)	−0.001 (5)	0.006 (5)	−0.005 (4)
C2A	0.024 (6)	0.012 (5)	0.015 (6)	−0.001 (4)	0.005 (5)	−0.001 (4)
C3A	0.015 (5)	0.022 (6)	0.013 (5)	0.002 (5)	0.008 (4)	−0.001 (4)
C4A	0.025 (6)	0.012 (5)	0.016 (5)	−0.002 (5)	0.007 (5)	0.000 (4)
C5A	0.018 (6)	0.015 (5)	0.015 (6)	−0.001 (5)	0.005 (4)	0.003 (4)
C6A	0.009 (5)	0.017 (5)	0.025 (6)	−0.003 (4)	0.010 (4)	−0.008 (5)
C7A	0.012 (6)	0.027 (7)	0.032 (7)	0.004 (5)	0.013 (5)	−0.010 (5)
C8A	0.018 (6)	0.029 (7)	0.020 (6)	0.005 (5)	0.007 (5)	0.000 (5)
C9A	0.039 (8)	0.019 (6)	0.025 (7)	0.000 (6)	0.000 (6)	−0.006 (5)
C10A	0.024 (6)	0.015 (5)	0.022 (6)	−0.006 (5)	0.004 (5)	−0.002 (5)
C11A	0.017 (6)	0.027 (6)	0.027 (6)	−0.011 (5)	0.014 (5)	−0.018 (5)
C12A	0.019 (6)	0.008 (5)	0.014 (5)	−0.003 (4)	0.006 (4)	−0.005 (4)
C13A	0.014 (4)	0.020 (5)	0.020 (5)	0.003 (4)	0.007 (4)	−0.003 (4)
C14A	0.031 (7)	0.021 (6)	0.012 (5)	0.002 (5)	0.003 (5)	0.004 (5)
C15A	0.019 (5)	0.023 (5)	0.017 (4)	0.002 (4)	0.013 (4)	0.001 (4)
C16A	0.038 (7)	0.017 (5)	0.008 (5)	0.002 (5)	0.009 (5)	−0.001 (4)
C17A	0.016 (6)	0.023 (6)	0.014 (5)	−0.005 (5)	0.005 (4)	−0.001 (5)
C18A	0.017 (6)	0.021 (6)	0.014 (5)	−0.003 (5)	0.011 (4)	−0.005 (4)
C19A	0.020 (6)	0.027 (6)	0.010 (5)	0.001 (5)	0.002 (4)	0.003 (5)
C20A	0.021 (6)	0.030 (6)	0.023 (6)	−0.006 (6)	0.000 (5)	−0.003 (5)
C21A	0.048 (9)	0.012 (5)	0.014 (6)	−0.008 (5)	0.008 (5)	−0.008 (5)
C22A	0.037 (8)	0.017 (6)	0.021 (6)	0.011 (6)	0.006 (6)	−0.001 (5)
C23A	0.024 (6)	0.018 (6)	0.020 (6)	−0.003 (5)	0.002 (5)	0.000 (5)
C24A	0.013 (4)	0.017 (4)	0.016 (4)	0.007 (4)	0.007 (4)	0.003 (4)
C25A	0.016 (5)	0.023 (5)	0.013 (4)	0.007 (4)	0.008 (4)	0.004 (4)
C26A	0.027 (6)	0.015 (5)	0.016 (5)	−0.001 (5)	0.006 (5)	−0.003 (4)
C27A	0.021 (6)	0.020 (6)	0.017 (6)	0.005 (5)	0.012 (5)	0.007 (5)
C28A	0.018 (6)	0.028 (6)	0.014 (5)	0.013 (5)	0.004 (5)	0.006 (5)
C29A	0.031 (7)	0.019 (6)	0.013 (5)	0.003 (5)	0.003 (5)	0.002 (5)
C30A	0.012 (5)	0.016 (5)	0.022 (6)	0.001 (4)	0.006 (4)	−0.001 (5)
C31A	0.030 (5)	0.012 (4)	0.019 (5)	−0.002 (4)	0.001 (4)	−0.006 (4)
C32A	0.043 (8)	0.018 (6)	0.026 (7)	0.003 (6)	0.001 (6)	−0.006 (5)
F1B	0.037 (5)	0.038 (4)	0.016 (3)	−0.002 (4)	−0.005 (3)	0.003 (3)
F2B	0.058 (6)	0.026 (4)	0.015 (3)	0.010 (4)	−0.001 (3)	0.004 (3)
O1B	0.036 (5)	0.016 (4)	0.017 (4)	0.002 (4)	0.010 (4)	−0.002 (3)
O2B	0.026 (5)	0.012 (4)	0.022 (4)	0.004 (3)	0.010 (3)	−0.002 (3)
N1B	0.020 (5)	0.011 (4)	0.013 (4)	0.002 (4)	0.002 (4)	0.001 (4)
N2B	0.022 (5)	0.018 (5)	0.015 (5)	0.002 (4)	0.006 (4)	−0.005 (4)
C1B	0.005 (5)	0.028 (6)	0.019 (6)	0.006 (4)	0.003 (4)	−0.007 (5)
C2B	0.010 (5)	0.023 (6)	0.016 (5)	−0.003 (4)	0.010 (4)	−0.007 (5)
C3B	0.018 (6)	0.012 (5)	0.023 (6)	−0.002 (4)	0.008 (5)	0.000 (4)
C4B	0.013 (4)	0.019 (5)	0.020 (5)	0.002 (4)	0.010 (4)	0.000 (4)
C5B	0.035 (7)	0.011 (5)	0.011 (5)	−0.002 (5)	0.011 (5)	−0.002 (4)
C6B	0.017 (4)	0.014 (4)	0.009 (3)	0.005 (3)	0.002 (3)	−0.001 (3)
C7B	0.024 (6)	0.015 (5)	0.011 (5)	0.000 (5)	0.003 (4)	−0.002 (4)
C8B	0.026 (7)	0.030 (7)	0.023 (6)	0.001 (6)	0.000 (5)	−0.010 (5)
C9B	0.015 (5)	0.026 (5)	0.025 (5)	−0.004 (4)	0.007 (4)	0.002 (4)
C10B	0.026 (5)	0.034 (5)	0.031 (5)	0.011 (5)	0.004 (4)	−0.010 (5)
C11B	0.014 (4)	0.020 (4)	0.012 (4)	0.003 (4)	0.003 (4)	−0.005 (4)
C12B	0.007 (5)	0.027 (6)	0.015 (5)	0.004 (5)	0.005 (4)	−0.002 (5)
C13B	0.039 (7)	0.014 (5)	0.015 (5)	−0.001 (5)	0.001 (5)	−0.002 (4)
C14B	0.041 (8)	0.018 (6)	0.011 (5)	0.006 (5)	0.000 (5)	−0.001 (5)
C15B	0.024 (6)	0.023 (6)	0.013 (5)	0.005 (5)	−0.004 (5)	−0.002 (5)
C16B	0.018 (6)	0.019 (6)	0.025 (6)	0.002 (5)	0.001 (5)	−0.002 (5)
C17B	0.025 (6)	0.007 (5)	0.019 (6)	−0.001 (4)	0.007 (5)	0.003 (4)
C18B	0.031 (7)	0.008 (5)	0.020 (6)	−0.002 (5)	0.003 (5)	0.001 (4)
C19B	0.023 (6)	0.023 (6)	0.023 (6)	0.006 (5)	0.012 (5)	−0.001 (5)
C20B	0.040 (8)	0.022 (6)	0.016 (6)	0.008 (6)	0.003 (5)	−0.007 (5)
C21B	0.010 (5)	0.022 (6)	0.036 (7)	0.003 (5)	0.001 (5)	−0.011 (5)
C22B	0.035 (8)	0.020 (6)	0.027 (7)	0.000 (6)	0.005 (6)	0.004 (5)
C23B	0.011 (5)	0.016 (5)	0.023 (6)	0.004 (4)	0.006 (5)	0.005 (5)
C24B	0.026 (6)	0.016 (5)	0.007 (5)	−0.006 (5)	0.006 (4)	0.002 (4)
C25B	0.010 (5)	0.027 (6)	0.013 (5)	−0.008 (5)	0.004 (4)	0.002 (5)
C26B	0.025 (6)	0.024 (6)	0.016 (6)	0.005 (5)	0.008 (5)	−0.001 (5)
C27B	0.026 (6)	0.019 (6)	0.015 (5)	−0.003 (5)	−0.004 (5)	0.004 (5)
C28B	0.022 (5)	0.021 (5)	0.012 (4)	−0.005 (4)	−0.001 (4)	0.009 (4)
C29B	0.022 (6)	0.018 (6)	0.017 (6)	−0.001 (5)	0.005 (5)	0.000 (5)
C30B	0.020 (6)	0.014 (5)	0.013 (5)	−0.001 (4)	0.008 (4)	0.002 (4)
C31B	0.011 (5)	0.016 (5)	0.027 (6)	0.002 (4)	0.007 (4)	−0.002 (5)
C32B	0.059 (10)	0.009 (5)	0.028 (7)	−0.004 (6)	0.005 (7)	−0.005 (5)

### Geometric parameters (Å, º)

**Table:**

F1A—C15A	1.362 (12)	F1B—C15B	1.370 (13)
F2A—C27A	1.347 (13)	F2B—C27B	1.361 (14)
O1A—C30A	1.242 (15)	O1B—C30B	1.235 (14)
O2A—C30A	1.352 (14)	O2B—C30B	1.343 (14)
O2A—C31A	1.461 (14)	O2B—C31B	1.442 (13)
N1A—C12A	1.407 (13)	N1B—C12B	1.423 (15)
N1A—C5A	1.466 (14)	N1B—C5B	1.465 (14)
N1A—C1A	1.485 (14)	N1B—C1B	1.496 (15)
N2A—C3A	1.383 (15)	N2B—C3B	1.354 (15)
N2A—C24A	1.432 (14)	N2B—C24B	1.407 (16)
N2A—H2AA	0.85 (3)	N2B—H2BA	0.8800
C1A—C6A	1.520 (17)	C1B—C6B	1.480 (15)
C1A—C2A	1.533 (16)	C1B—C2B	1.540 (15)
C1A—H1AA	1.0000	C1B—H1BA	1.0000
C2A—C3A	1.366 (16)	C2B—C3B	1.347 (17)
C2A—C30A	1.423 (16)	C2B—C30B	1.454 (15)
C3A—C4A	1.484 (16)	C3B—C4B	1.523 (15)
C4A—C5A	1.532 (15)	C4B—C5B	1.539 (18)
C4A—H4AA	0.9900	C4B—H4BA	0.9900
C4A—H4AB	0.9900	C4B—H4BB	0.9900
C5A—C18A	1.524 (16)	C5B—C18B	1.529 (15)
C5A—H5AA	1.0000	C5B—H5BA	1.0000
C6A—C7A	1.402 (15)	C6B—C11B	1.395 (14)
C6A—C11A	1.436 (17)	C6B—C7B	1.451 (16)
C7A—C8A	1.372 (18)	C7B—C8B	1.347 (18)
C7A—H7AA	0.9500	C7B—H7BA	0.9500
C8A—C9A	1.406 (19)	C8B—C9B	1.405 (17)
C8A—H8AA	0.9500	C8B—H8BA	0.9500
C9A—C10A	1.399 (17)	C9B—C10B	1.430 (19)
C9A—H9AA	0.9500	C9B—H9BA	0.9500
C10A—C11A	1.383 (19)	C10B—C11B	1.340 (18)
C10A—H10A	0.9500	C10B—H10B	0.9500
C11A—H11A	0.9500	C11B—H11B	0.9500
C12A—C17A	1.392 (16)	C12B—C17B	1.382 (16)
C12A—C13A	1.413 (16)	C12B—C13B	1.398 (16)
C13A—C14A	1.393 (16)	C13B—C14B	1.397 (18)
C13A—H13A	0.9500	C13B—H13B	0.9500
C14A—C15A	1.370 (18)	C14B—C15B	1.373 (19)
C14A—H14A	0.9500	C14B—H14B	0.9500
C15A—C16A	1.367 (16)	C15B—C16B	1.371 (17)
C16A—C17A	1.392 (15)	C16B—C17B	1.414 (17)
C16A—H16A	0.9500	C16B—H16B	0.9500
C17A—H17A	0.9500	C17B—H17B	0.9500
C18A—C23A	1.406 (17)	C18B—C19B	1.365 (16)
C18A—C19A	1.413 (17)	C18B—C23B	1.395 (16)
C19A—C20A	1.389 (18)	C19B—C20B	1.394 (17)
C19A—H19A	0.9500	C19B—H19B	0.9500
C20A—C21A	1.415 (19)	C20B—C21B	1.384 (18)
C20A—H20A	0.9500	C20B—H20B	0.9500
C21A—C22A	1.387 (19)	C21B—C22B	1.411 (18)
C21A—H21A	0.9500	C21B—H21B	0.9500
C22A—C23A	1.370 (18)	C22B—C23B	1.390 (18)
C22A—H22A	0.9500	C22B—H22B	0.9500
C23A—H23A	0.9500	C23B—H23B	0.9500
C24A—C25A	1.359 (17)	C24B—C25B	1.362 (17)
C24A—C29A	1.413 (17)	C24B—C29B	1.409 (15)
C25A—C26A	1.396 (16)	C25B—C26B	1.373 (17)
C25A—H25A	0.9500	C25B—H25B	0.9500
C26A—C27A	1.382 (17)	C26B—C27B	1.386 (16)
C26A—H26A	0.9500	C26B—H26B	0.9500
C27A—C28A	1.352 (18)	C27B—C28B	1.349 (18)
C28A—C29A	1.412 (16)	C28B—C29B	1.409 (17)
C28A—H28A	0.9500	C28B—H28B	0.9500
C29A—H29A	0.9500	C29B—H29B	0.9500
C31A—C32A	1.498 (18)	C31B—C32B	1.509 (18)
C31A—H31A	0.9900	C31B—H31C	0.9900
C31A—H31B	0.9900	C31B—H31D	0.9900
C32A—H32A	0.9800	C32B—H32D	0.9800
C32A—H32B	0.9800	C32B—H32E	0.9800
C32A—H32C	0.9800	C32B—H32F	0.9800
			
C30A—O2A—C31A	116.6 (9)	C30B—O2B—C31B	115.9 (8)
C12A—N1A—C5A	117.6 (9)	C12B—N1B—C5B	118.2 (10)
C12A—N1A—C1A	115.0 (9)	C12B—N1B—C1B	116.3 (9)
C5A—N1A—C1A	118.2 (8)	C5B—N1B—C1B	116.8 (9)
C3A—N2A—C24A	125.0 (11)	C3B—N2B—C24B	127.3 (10)
C3A—N2A—H2AA	116 (9)	C3B—N2B—H2BA	116.4
C24A—N2A—H2AA	118 (9)	C24B—N2B—H2BA	116.4
N1A—C1A—C6A	111.2 (10)	C6B—C1B—N1B	112.5 (9)
N1A—C1A—C2A	111.8 (10)	C6B—C1B—C2B	112.6 (10)
C6A—C1A—C2A	113.9 (9)	N1B—C1B—C2B	111.4 (10)
N1A—C1A—H1AA	106.5	C6B—C1B—H1BA	106.7
C6A—C1A—H1AA	106.5	N1B—C1B—H1BA	106.7
C2A—C1A—H1AA	106.5	C2B—C1B—H1BA	106.7
C3A—C2A—C30A	121.5 (11)	C3B—C2B—C30B	121.2 (10)
C3A—C2A—C1A	116.1 (10)	C3B—C2B—C1B	118.5 (10)
C30A—C2A—C1A	122.3 (10)	C30B—C2B—C1B	120.3 (10)
C2A—C3A—N2A	121.6 (11)	C2B—C3B—N2B	125.7 (10)
C2A—C3A—C4A	117.3 (10)	C2B—C3B—C4B	114.5 (10)
N2A—C3A—C4A	120.7 (10)	N2B—C3B—C4B	119.3 (10)
C3A—C4A—C5A	108.2 (9)	C3B—C4B—C5B	109.2 (10)
C3A—C4A—H4AA	110.1	C3B—C4B—H4BA	109.8
C5A—C4A—H4AA	110.1	C5B—C4B—H4BA	109.8
C3A—C4A—H4AB	110.1	C3B—C4B—H4BB	109.8
C5A—C4A—H4AB	110.1	C5B—C4B—H4BB	109.8
H4AA—C4A—H4AB	108.4	H4BA—C4B—H4BB	108.3
N1A—C5A—C18A	111.5 (9)	N1B—C5B—C18B	112.9 (9)
N1A—C5A—C4A	108.2 (9)	N1B—C5B—C4B	109.3 (10)
C18A—C5A—C4A	108.5 (9)	C18B—C5B—C4B	108.1 (10)
N1A—C5A—H5AA	109.5	N1B—C5B—H5BA	108.8
C18A—C5A—H5AA	109.5	C18B—C5B—H5BA	108.8
C4A—C5A—H5AA	109.5	C4B—C5B—H5BA	108.8
C7A—C6A—C11A	115.2 (11)	C11B—C6B—C7B	113.9 (10)
C7A—C6A—C1A	122.8 (11)	C11B—C6B—C1B	123.2 (10)
C11A—C6A—C1A	121.5 (10)	C7B—C6B—C1B	122.7 (10)
C8A—C7A—C6A	123.2 (13)	C8B—C7B—C6B	122.5 (10)
C8A—C7A—H7AA	118.4	C8B—C7B—H7BA	118.7
C6A—C7A—H7AA	118.4	C6B—C7B—H7BA	118.7
C7A—C8A—C9A	120.7 (12)	C7B—C8B—C9B	121.5 (12)
C7A—C8A—H8AA	119.7	C7B—C8B—H8BA	119.2
C9A—C8A—H8AA	119.7	C9B—C8B—H8BA	119.2
C10A—C9A—C8A	118.1 (13)	C8B—C9B—C10B	116.8 (11)
C10A—C9A—H9AA	120.9	C8B—C9B—H9BA	121.6
C8A—C9A—H9AA	120.9	C10B—C9B—H9BA	121.6
C11A—C10A—C9A	120.7 (12)	C11B—C10B—C9B	120.4 (11)
C11A—C10A—H10A	119.6	C11B—C10B—H10B	119.8
C9A—C10A—H10A	119.6	C9B—C10B—H10B	119.8
C10A—C11A—C6A	121.9 (11)	C10B—C11B—C6B	124.8 (12)
C10A—C11A—H11A	119.0	C10B—C11B—H11B	117.6
C6A—C11A—H11A	119.0	C6B—C11B—H11B	117.6
C17A—C12A—N1A	121.4 (10)	C17B—C12B—C13B	117.8 (11)
C17A—C12A—C13A	119.6 (10)	C17B—C12B—N1B	123.4 (11)
N1A—C12A—C13A	118.7 (10)	C13B—C12B—N1B	118.7 (11)
C14A—C13A—C12A	119.5 (11)	C14B—C13B—C12B	121.7 (12)
C14A—C13A—H13A	120.3	C14B—C13B—H13B	119.2
C12A—C13A—H13A	120.3	C12B—C13B—H13B	119.2
C15A—C14A—C13A	118.7 (11)	C15B—C14B—C13B	118.8 (11)
C15A—C14A—H14A	120.7	C15B—C14B—H14B	120.6
C13A—C14A—H14A	120.7	C13B—C14B—H14B	120.6
F1A—C15A—C16A	118.9 (11)	F1B—C15B—C16B	119.4 (11)
F1A—C15A—C14A	117.7 (11)	F1B—C15B—C14B	119.1 (11)
C16A—C15A—C14A	123.4 (10)	C16B—C15B—C14B	121.5 (11)
C15A—C16A—C17A	118.4 (11)	C15B—C16B—C17B	119.1 (11)
C15A—C16A—H16A	120.8	C15B—C16B—H16B	120.4
C17A—C16A—H16A	120.8	C17B—C16B—H16B	120.4
C16A—C17A—C12A	120.3 (11)	C12B—C17B—C16B	121.0 (11)
C16A—C17A—H17A	119.8	C12B—C17B—H17B	119.5
C12A—C17A—H17A	119.8	C16B—C17B—H17B	119.5
C23A—C18A—C19A	116.8 (11)	C19B—C18B—C23B	118.7 (11)
C23A—C18A—C5A	118.1 (10)	C19B—C18B—C5B	123.7 (10)
C19A—C18A—C5A	124.8 (11)	C23B—C18B—C5B	117.5 (10)
C20A—C19A—C18A	122.6 (11)	C18B—C19B—C20B	119.7 (12)
C20A—C19A—H19A	118.7	C18B—C19B—H19B	120.2
C18A—C19A—H19A	118.7	C20B—C19B—H19B	120.2
C19A—C20A—C21A	118.5 (11)	C21B—C20B—C19B	122.8 (11)
C19A—C20A—H20A	120.7	C21B—C20B—H20B	118.6
C21A—C20A—H20A	120.7	C19B—C20B—H20B	118.6
C22A—C21A—C20A	119.1 (11)	C20B—C21B—C22B	117.6 (11)
C22A—C21A—H21A	120.4	C20B—C21B—H21B	121.2
C20A—C21A—H21A	120.4	C22B—C21B—H21B	121.2
C23A—C22A—C21A	121.8 (12)	C23B—C22B—C21B	119.0 (12)
C23A—C22A—H22A	119.1	C23B—C22B—H22B	120.5
C21A—C22A—H22A	119.1	C21B—C22B—H22B	120.5
C22A—C23A—C18A	121.1 (12)	C22B—C23B—C18B	122.2 (11)
C22A—C23A—H23A	119.5	C22B—C23B—H23B	118.9
C18A—C23A—H23A	119.5	C18B—C23B—H23B	118.9
C25A—C24A—C29A	122.0 (10)	C25B—C24B—N2B	124.4 (10)
C25A—C24A—N2A	123.1 (11)	C25B—C24B—C29B	119.0 (11)
C29A—C24A—N2A	114.5 (11)	N2B—C24B—C29B	116.4 (10)
C24A—C25A—C26A	119.5 (11)	C24B—C25B—C26B	121.3 (10)
C24A—C25A—H25A	120.2	C24B—C25B—H25B	119.4
C26A—C25A—H25A	120.2	C26B—C25B—H25B	119.4
C27A—C26A—C25A	118.3 (12)	C25B—C26B—C27B	119.8 (11)
C27A—C26A—H26A	120.8	C25B—C26B—H26B	120.1
C25A—C26A—H26A	120.8	C27B—C26B—H26B	120.1
F2A—C27A—C28A	118.5 (11)	C28B—C27B—F2B	120.7 (10)
F2A—C27A—C26A	118.1 (11)	C28B—C27B—C26B	120.8 (11)
C28A—C27A—C26A	123.2 (11)	F2B—C27B—C26B	118.5 (11)
C27A—C28A—C29A	119.2 (11)	C27B—C28B—C29B	119.8 (10)
C27A—C28A—H28A	120.4	C27B—C28B—H28B	120.1
C29A—C28A—H28A	120.4	C29B—C28B—H28B	120.1
C28A—C29A—C24A	117.5 (11)	C28B—C29B—C24B	119.3 (11)
C28A—C29A—H29A	121.3	C28B—C29B—H29B	120.4
C24A—C29A—H29A	121.3	C24B—C29B—H29B	120.4
O1A—C30A—O2A	120.4 (10)	O1B—C30B—O2B	123.0 (10)
O1A—C30A—C2A	126.4 (11)	O1B—C30B—C2B	122.9 (11)
O2A—C30A—C2A	113.1 (10)	O2B—C30B—C2B	114.1 (9)
O2A—C31A—C32A	110.8 (10)	O2B—C31B—C32B	111.0 (10)
O2A—C31A—H31A	109.5	O2B—C31B—H31C	109.4
C32A—C31A—H31A	109.5	C32B—C31B—H31C	109.4
O2A—C31A—H31B	109.5	O2B—C31B—H31D	109.4
C32A—C31A—H31B	109.5	C32B—C31B—H31D	109.4
H31A—C31A—H31B	108.1	H31C—C31B—H31D	108.0
C31A—C32A—H32A	109.5	C31B—C32B—H32D	109.5
C31A—C32A—H32B	109.5	C31B—C32B—H32E	109.5
H32A—C32A—H32B	109.5	H32D—C32B—H32E	109.5
C31A—C32A—H32C	109.5	C31B—C32B—H32F	109.5
H32A—C32A—H32C	109.5	H32D—C32B—H32F	109.5
H32B—C32A—H32C	109.5	H32E—C32B—H32F	109.5
			
C12A—N1A—C1A—C6A	109.5 (11)	C12B—N1B—C1B—C6B	110.8 (11)
C5A—N1A—C1A—C6A	−104.3 (12)	C5B—N1B—C1B—C6B	−102.2 (12)
C12A—N1A—C1A—C2A	−122.0 (11)	C12B—N1B—C1B—C2B	−121.7 (10)
C5A—N1A—C1A—C2A	24.2 (15)	C5B—N1B—C1B—C2B	25.2 (13)
N1A—C1A—C2A—C3A	−43.2 (14)	C6B—C1B—C2B—C3B	80.7 (14)
C6A—C1A—C2A—C3A	83.8 (13)	N1B—C1B—C2B—C3B	−46.7 (14)
N1A—C1A—C2A—C30A	138.3 (11)	C6B—C1B—C2B—C30B	−99.2 (12)
C6A—C1A—C2A—C30A	−94.6 (13)	N1B—C1B—C2B—C30B	133.4 (11)
C30A—C2A—C3A—N2A	−2.1 (18)	C30B—C2B—C3B—N2B	1.5 (19)
C1A—C2A—C3A—N2A	179.4 (11)	C1B—C2B—C3B—N2B	−178.4 (11)
C30A—C2A—C3A—C4A	−175.2 (11)	C30B—C2B—C3B—C4B	−170.2 (10)
C1A—C2A—C3A—C4A	6.3 (16)	C1B—C2B—C3B—C4B	9.9 (15)
C24A—N2A—C3A—C2A	160.7 (11)	C24B—N2B—C3B—C2B	160.3 (12)
C24A—N2A—C3A—C4A	−26.4 (18)	C24B—N2B—C3B—C4B	−28.4 (18)
C2A—C3A—C4A—C5A	47.0 (14)	C2B—C3B—C4B—C5B	44.4 (13)
N2A—C3A—C4A—C5A	−126.2 (12)	N2B—C3B—C4B—C5B	−127.8 (12)
C12A—N1A—C5A—C18A	−68.8 (13)	C12B—N1B—C5B—C18B	−67.5 (13)
C1A—N1A—C5A—C18A	145.9 (10)	C1B—N1B—C5B—C18B	146.2 (10)
C12A—N1A—C5A—C4A	171.9 (10)	C12B—N1B—C5B—C4B	172.2 (9)
C1A—N1A—C5A—C4A	26.6 (14)	C1B—N1B—C5B—C4B	25.9 (13)
C3A—C4A—C5A—N1A	−62.8 (12)	C3B—C4B—C5B—N1B	−62.7 (11)
C3A—C4A—C5A—C18A	176.1 (10)	C3B—C4B—C5B—C18B	174.1 (9)
N1A—C1A—C6A—C7A	157.8 (11)	N1B—C1B—C6B—C11B	−27.8 (15)
C2A—C1A—C6A—C7A	30.4 (15)	C2B—C1B—C6B—C11B	−154.6 (11)
N1A—C1A—C6A—C11A	−30.9 (15)	N1B—C1B—C6B—C7B	158.7 (10)
C2A—C1A—C6A—C11A	−158.3 (11)	C2B—C1B—C6B—C7B	31.9 (15)
C11A—C6A—C7A—C8A	3.5 (18)	C11B—C6B—C7B—C8B	1.8 (17)
C1A—C6A—C7A—C8A	175.3 (12)	C1B—C6B—C7B—C8B	175.8 (12)
C6A—C7A—C8A—C9A	−4 (2)	C6B—C7B—C8B—C9B	−3 (2)
C7A—C8A—C9A—C10A	2.5 (19)	C7B—C8B—C9B—C10B	4.3 (19)
C8A—C9A—C10A—C11A	−1.3 (18)	C8B—C9B—C10B—C11B	−4.5 (19)
C9A—C10A—C11A—C6A	1.4 (18)	C9B—C10B—C11B—C6B	4 (2)
C7A—C6A—C11A—C10A	−2.3 (17)	C7B—C6B—C11B—C10B	−2.1 (17)
C1A—C6A—C11A—C10A	−174.2 (11)	C1B—C6B—C11B—C10B	−176.1 (13)
C5A—N1A—C12A—C17A	−1.1 (16)	C5B—N1B—C12B—C17B	−5.6 (15)
C1A—N1A—C12A—C17A	145.3 (11)	C1B—N1B—C12B—C17B	140.9 (11)
C5A—N1A—C12A—C13A	172.6 (10)	C5B—N1B—C12B—C13B	171.2 (10)
C1A—N1A—C12A—C13A	−41.0 (15)	C1B—N1B—C12B—C13B	−42.3 (13)
C17A—C12A—C13A—C14A	−2.0 (17)	C17B—C12B—C13B—C14B	−0.9 (17)
N1A—C12A—C13A—C14A	−175.7 (11)	N1B—C12B—C13B—C14B	−177.8 (10)
C12A—C13A—C14A—C15A	1.2 (19)	C12B—C13B—C14B—C15B	1.5 (18)
C13A—C14A—C15A—F1A	179.4 (11)	C13B—C14B—C15B—F1B	178.9 (10)
C13A—C14A—C15A—C16A	−1 (2)	C13B—C14B—C15B—C16B	−1.9 (18)
F1A—C15A—C16A—C17A	−178.7 (11)	F1B—C15B—C16B—C17B	−179.2 (10)
C14A—C15A—C16A—C17A	2 (2)	C14B—C15B—C16B—C17B	1.6 (18)
C15A—C16A—C17A—C12A	−2.7 (18)	C13B—C12B—C17B—C16B	0.6 (16)
N1A—C12A—C17A—C16A	176.3 (11)	N1B—C12B—C17B—C16B	177.4 (10)
C13A—C12A—C17A—C16A	2.8 (17)	C15B—C16B—C17B—C12B	−1.0 (17)
N1A—C5A—C18A—C23A	160.1 (10)	N1B—C5B—C18B—C19B	−22.1 (18)
C4A—C5A—C18A—C23A	−80.8 (13)	C4B—C5B—C18B—C19B	98.9 (14)
N1A—C5A—C18A—C19A	−25.1 (15)	N1B—C5B—C18B—C23B	159.1 (11)
C4A—C5A—C18A—C19A	94.0 (12)	C4B—C5B—C18B—C23B	−79.9 (13)
C23A—C18A—C19A—C20A	−3.1 (16)	C23B—C18B—C19B—C20B	0.8 (19)
C5A—C18A—C19A—C20A	−178.0 (11)	C5B—C18B—C19B—C20B	−178.0 (13)
C18A—C19A—C20A—C21A	2.6 (17)	C18B—C19B—C20B—C21B	0 (2)
C19A—C20A—C21A—C22A	−0.3 (18)	C19B—C20B—C21B—C22B	−2 (2)
C20A—C21A—C22A—C23A	−1.4 (19)	C20B—C21B—C22B—C23B	3 (2)
C21A—C22A—C23A—C18A	0.8 (19)	C21B—C22B—C23B—C18B	−2 (2)
C19A—C18A—C23A—C22A	1.4 (16)	C19B—C18B—C23B—C22B	0.1 (19)
C5A—C18A—C23A—C22A	176.6 (11)	C5B—C18B—C23B—C22B	178.9 (13)
C3A—N2A—C24A—C25A	−32.1 (18)	C3B—N2B—C24B—C25B	−31.2 (19)
C3A—N2A—C24A—C29A	154.7 (11)	C3B—N2B—C24B—C29B	153.9 (11)
C29A—C24A—C25A—C26A	−5.9 (17)	N2B—C24B—C25B—C26B	−178.5 (11)
N2A—C24A—C25A—C26A	−178.6 (10)	C29B—C24B—C25B—C26B	−3.8 (18)
C24A—C25A—C26A—C27A	4.7 (17)	C24B—C25B—C26B—C27B	1.6 (19)
C25A—C26A—C27A—F2A	−177.9 (10)	C25B—C26B—C27B—C28B	1.4 (19)
C25A—C26A—C27A—C28A	−1.9 (18)	C25B—C26B—C27B—F2B	−178.5 (11)
F2A—C27A—C28A—C29A	176.1 (10)	F2B—C27B—C28B—C29B	177.8 (10)
C26A—C27A—C28A—C29A	0.1 (18)	C26B—C27B—C28B—C29B	−2.1 (19)
C27A—C28A—C29A—C24A	−1.1 (17)	C27B—C28B—C29B—C24B	−0.2 (18)
C25A—C24A—C29A—C28A	4.1 (17)	C25B—C24B—C29B—C28B	3.1 (17)
N2A—C24A—C29A—C28A	177.4 (10)	N2B—C24B—C29B—C28B	178.2 (10)
C31A—O2A—C30A—O1A	−1.6 (15)	C31B—O2B—C30B—O1B	1.5 (17)
C31A—O2A—C30A—C2A	176.8 (10)	C31B—O2B—C30B—C2B	−179.9 (10)
C3A—C2A—C30A—O1A	3.2 (19)	C3B—C2B—C30B—O1B	2.1 (19)
C1A—C2A—C30A—O1A	−178.4 (12)	C1B—C2B—C30B—O1B	−178.0 (11)
C3A—C2A—C30A—O2A	−175.1 (11)	C3B—C2B—C30B—O2B	−176.4 (11)
C1A—C2A—C30A—O2A	3.3 (15)	C1B—C2B—C30B—O2B	3.4 (15)
C30A—O2A—C31A—C32A	−82.5 (13)	C30B—O2B—C31B—C32B	−82.7 (12)

### Hydrogen-bond geometry (Å, º)

**Table:**

*D*—H···*A*	*D*—H	H···*A*	*D*···*A*	*D*—H···*A*
N2*A*—H2*AA*···O1*A*	0.85 (3)	1.98 (10)	2.652 (13)	136 (12)
N2*B*—H2*BA*···O1*B*	0.88	2.04	2.664 (13)	127
C31*A*—H31*A*···F2*B*^i^	0.99	2.43	3.327 (14)	151
C31*A*—H31*B*···F1*B*^ii^	0.99	2.40	3.257 (15)	144
C31*B*—H31*C*···F2*A*^iii^	0.99	2.45	3.213 (15)	134
C31*B*—H31*D*···F1*A*^iv^	0.99	2.32	3.281 (13)	165
C5*A*—H5*AA*···*Cg*9^v^	1.00	2.91	3.907 (13)	178
C5*B*—H5*BA*···*Cg*4^vi^	1.00	2.96	3.958 (14)	174
C22*A*—H22*A*···*Cg*7^v^	0.95	2.87	3.770 (14)	159
C22*B*—H22*B*···*Cg*2^vi^	0.95	2.94	3.857 (15)	162
C29*A*—H29*A*···*Cg*7^vii^	0.95	2.71	3.437 (13)	134
C29*B*—H29*B*···*Cg*2^viii^	0.95	2.84	3.595 (13)	138

Symmetry codes: (i) −*x*+1, *y*+1/2, −*z*; (ii) −*x*+2, *y*+1/2, −*z*+1; (iii) −*x*+1, *y*−1/2, −*z*+1; (iv) −*x*+2, *y*−1/2, −*z*; (v) −*x*+1, *y*−1/2, −*z*; (vi) −*x*, *y*+1/2, −*z*; (vii) *x*−1, *y*, *z*+1; (viii) *x*+1, *y*, *z*.

### Comparison of selected (X-ray and DFT) bond lengths and angles (Å, °).

**Table:**

Bonds/angles	X-ray	B3LYP/6-311+G(2d,p)
F1A—C15A	1.363 (12)	1.3636
F2A—C27A	1.346 (13)	1.3463
O1A—C30A	1.242 (15)	1.2407
O2A—C30A	1.351 (14)	1.3509
O2A—C31A	1.464 (14)	1.4637
N1A—C12A	1.408 (13)	1.4078
N1A—C5A	1.467 (14)	1.466
N1A—C1A	1.485 (14)	1.4854
N2A—C24A	1.431 (14)	1.4318
		
C30A—O2—C31A	116.6 (9)	116.5807
C12A—N1A—C1A	115.1 (9)	115.088
C5A—N1A—C1A	118.2 (8)	117.6837
C3A—N2A—C24A	125.0 (11)	124.9578
N1A—C1A—C6A	111.1 (10)	111.1474
N1A—C1A—C2A	111.9 (10)	111.8615
F1A—C15A—C14A	117.7 (11)	118.7741
F1A—C15A—C16A	118.8 (11)	119.0320
F2A—C27A—C28A	118.8 (11)	119.5501
F2A—C27A—C26A	118.1 (11)	119.3655
O1A—C30A –O2A	120.4 (10)	120.4296
O1A—C30A)—C2A	126.4 (11)	126.4327
O2A—C30A –C2A	113.2 (10)	113.1168
O2A—C31A –C32A	110.7 (10)	110.7128

### Calculated energies

**Table:**

Molecular property	Title compound
Total energy, TE (eV)	-46146
*E*_HOMO_	-5.6182
*E*_LUMO_	-1.3986
Gap, Δ*E* (eV)	4.22
Dipole moment, µ (Debye)	3.5082
Ionization enthalpy, IE (eV)	5.6182
Electron gain enthalpy, EE (eV)	1.3986
Electronegativity, χ	3.508
Hardness, η	2.1098
Softness, σ	0.2369
Electrophilicity index, ω	2.9167

### Second-order perturbation theory analysis of Fock matrix in NBO basis for (I)

**Table:**

NBO	Donor	Occupancy	NBO	Acceptor	Occupancy	*E*(2)*^a^*	*E*(*j*) –	*F*(*ij*)*^c^*
No.			No.			(kcal	*E*(*i*)*^b^*	(a.u.)
						mol^-1^)	(a.u.)	(a.u.)
59	π(C44-C53)	1.64436	1170	π*(C45-C47)	0.35828	19.09	0.28	0.065
59	π(C44-C53)	1.64436	1175	π*(C49-C51)	0.38366	19.14	0.31	0.069
45	π(C33-C42)	1.66035	1156	π*(C34-C36)	0.32171	17.47	0.32	0.067
45	π(C33-C42)	1.66035	1161	π*(C38-C40)	0.33276	20.75	0.28	0.069
19	π(C7-C8)	1.65789	1133	π*(C10-C12)	0.37463	19.76	0.28	0.067
19	π(C7-C8)	1.65789	1138	π*(C14-C16)	0.33955	20.06	0.29	0.068
72	π(C56-C57)	1.65426	1186	π*(C59-C61)	0.33151	19.54	0.30	0.069
53	π(C38-C40)	1.66153	1153	π*(C33-C42)	0.35511	19.12	0.29	0.067
53	π(C38-C40)	1.66153	1156	π*(C34-C36)	0.32171	18.31	0.32	0.068
48	π(C34-C36)	1.65591	1161	π*(C38-C40)	0.33276	20.46	0.28	0.068
48	π(C34-C36)	1.65591	1153	π*(C33-C42)	0.35511	21.06	0.29	0.070
30	π(C14-C16)	1.69306	1133	π*(C11-C13)	0.37463	21.43	0.28	0.070
30	π(C14-C16)	1.69306	1127	π*(C8-C9)	0.39217	19.23	0.28	0.067
25	π(C10-C12)	1.65797	1138	π*(C15-C17)	0.33955	19.96	0.29	0.068
25	π(C10-C12)	1.65797	1127	π*(C8-C9)	0.39217	20.13	0.29	0.069
15	π(C5-C19)	1.79891	1142	π*(C20-O21)	0.36217	30.34	0.27	0.084
62	π(C45-C47)	1.72021	1167	π*(C44-C53)	0.40601	18.64	0.29	0.068
62	π(C45-C47)	1.72021	1175	π*(C49-C51)	0.38366	17.53	0.31	0.068
67	π(C49-C51)	1.67609	1167	π*(C44-C53)	0.40601	17.81	0.29	0.066
67	π(C49-C51)	1.67609	1170	π*(C45-C47)	0.35828	21.20	0.29	0.071
73	π(C56-C65)	1.97102	1191	π*(C63-C65)	0.32811	14.60	1.98	0.152
78	π(C59-C61)	1.66853	1180	π*(C56-C57)	0.34323	15.72	0.34	0.066
83	π(C63-C65)	1.67036	1180	π*(C56-C57)	0.34323	13.27	0.34	0.061
83	π(C63-C65)	1.67036	1186	π*(C59-C61)	0.33151	16.27	0.30	0.063
124	(LP1-N6)	1.64338	1123	π*(C5-C19)	0.30393	54.49	0.29	0.114
124	(LP1-N6)	1.64338	1127	π*(C8-C9)	0.39217	20.26	0.27	0.067
130	(LP1-O22)	1.96095	1142	σ*(C20-21)	0.36217	7.89	1.13	0.085
127	(LP2-F13)	1.92911	1133	π*(C11-C13)	0.37463	18.00	0.43	0.085
126	(LP2-F13)	1.97237	1133	σ*(C11-C13)	0.37463	6.04	0.97	0.068
133	(LP2-F50)	1.97282	1175	σ*(C49-C51)	0.38366	5.94	0.98	0.068
131	(LP2-O22)	1.80316	1142	π*(C20-O21)	0.36217	46.33	0.32	0.114
134	(LP3-F50)	1.93507	1175	π*(C49-C51)	0.38366	15.94	0.46	0.084

Notes: (*a*) (2) means energy of hyperconjugative interactions; (*b*) energy difference between donor and accepter *i* and *j* NBO orbitals; (*c*) F(*i*,*j*) is the Fock matrix element between *i* and *j* NBO orbitals
